# The Prognostic Significance of Tumor Budding and Cell Nest Size in Laryngeal Squamous Cell Carcinoma

**DOI:** 10.1002/cnr2.70052

**Published:** 2024-11-26

**Authors:** Kiana Anousha, Mohammad Moin Shekari, Elham Mirzaian, Tahere Yousefi, Amin Beheshti, Maryam Lotfi

**Affiliations:** ^1^ Pathology Department Tehran University of Medical Sciences Tehran Iran; ^2^ Pathology Department Shariati Hospital, Tehran University of Medical Sciences Tehran Iran; ^3^ Pathology Department Amir Alam Hospital, Tehran University of Medical Sciences Tehran Iran; ^4^ Otorhinolaryngology Research Center Amir Alam Hospital, Tehran University of Medical Sciences Tehran Iran

**Keywords:** cell nest size, larynx, squamous cell carcinoma, tumor budding

## Abstract

**Background:**

Laryngeal squamous cell carcinoma (LSCC) is a commonly occurring malignancy in the head and neck region. However, due to the heterogeneity of primary tumor sites, tumor behavior, and molecular mechanisms, there is currently no consensus on the accuracy of clinicopathological prognostic factors for individual cases. Tumor histopathologic behavior remains a crucial factor in predicting aggressiveness. Recent studies have shown that peritumoral tumor budding (TB) combined with cell nest size (CNS) is a reliable marker for predicting lymph node metastasis, advanced cancer prognosis, and therapeutic response in SCCs of different origins.

**Aims:**

This study aims to investigate the relationship between TB and CNS in the context of nodal metastasis and overall prognosis in patients diagnosed with LSCC. Our objective is to establish the significance of TB and CNS status as a cost‐effective, easily assessed, and highly reliable prognostic factor among this patient population.

**Methods and results:**

In this retrospective cross‐sectional study, we analyzed 128 LSCC cases that underwent total laryngectomy at Amir Alam Hospital. We evaluated TB and CNS based on the Boxberg et al. study. Our study demonstrated a significant correlation between TB, and nodal involvement (*p* = 0.015), vascular invasion (*p* = 0.035), and mortality rate (*p* = 0.001), as well as a significant statistical correlation between high TB and extra‐laryngeal extension (*p* = 0.006), clinical stage (*p* = 0.011), and mortality rate (*p* = 0.001). Moreover, small nest size was also associated with the clinical stage (*p* = 0.047), extra‐laryngeal extension (*p* = 0.015), and mortality rate (*p* < 0.001). Based on our results, TB, CNS, and clinical stage are independent prognostic factors for mortality rate and are correlated with disease‐specific survival.

**Conclusion:**

Given the effect of TB and CNS on the overall prognosis and survival of patients with LSCC, evaluating these two factors on routine H&E microscopic examination of LSCC specimens is recommended to facilitate individualized risk assessment and treatment planning.

## 1 Introduction

Laryngeal squamous cell carcinoma (LSCC) originates from the mucosal epithelium of the larynx and is among the most frequently occurring malignancies in the head and neck region. Unfortunately, the incidence of LSCC, along with other head and neck SCCs, has been steadily increasing. According to GLOBACAN, the Global Cancer Observatory, there is an anticipated 30% rise in the number of cancer cases by the year 2030 [1]. This rise may be due to several factors, including a lack of concrete screening strategies, carcinogen consumption, and increased human papillomavirus (HPV) infections [2]. Despite the increase in cases, the survival rate of LSCCs has not significantly decreased over the past few decades. On average, the 5‐year survival rate is about 40%–60%, regardless of the patient's age or anatomical site of the tumor [3]. Interestingly, some studies suggest that the slight improvement in survival is more related to the rise of patients with HPV‐associated LSCC, which has a better overall outcome than actual advances in cancer control [2].

The prognostic factors of LSCC are typically grouped into three categories: host, tumor, and treatment, which encompass various elements such as age, gender, nutritional or performance status, tumor site, tumor histological grading, perineural/vascular invasion, and TNM staging [4]. Despite this, studies have shown conflicting results regarding their accuracy in predicting patient outcomes [5]. As a result, there is currently no definitive predictor of survival due to the primary tumor site's heterogeneity, tumor behavior, and molecular mechanisms [6]. For this reason, quests for discovering a more reliable prognostic parameter have been carried out.

When predicting tumor behavior and assessing its aggressiveness, it is important to consider the histopathologic findings. Tumor progression and response to treatment are also closely linked to how tumor cells interact with the microenvironment around them [7]. Studies have shown that cancer cells at the tumor front tend to be more aggressive and contribute to tumor growth [8]. Peritumoral tumor budding (TB) occurs when small clusters of non‐glandular cancer cells appear throughout the invasive tumor front. This is associated with decreased cell adhesion and increased local invasion [9]. While TB is commonly reported in colorectal cancers, it is now recognized as a reliable predictor of lymph node metastasis, advanced cancer prognosis, and response to therapy in multiple malignancies such as lung SCC, esophageal SCC, breast cancer, and pancreatic ductal adenocarcinoma [10, 11]. The TBNS (tumor budding/nest size) grading system, which combines TB with cell nest size (CNS), is also considered a practical prognostic element in SCCs of various sites, including lung, oral cavity, larynx, and hypopharynx [12, 13]. The adverse effect of TB on survival of LSCC, even in patients with a lower TNM staging score, have also been confirmed in previous studies [14, 15, 16]. Despite the significant impact TB can have on patient survival, it has yet to be included in standard pathology reports for LSCC due to limited data and a lack of systematic scoring systems.

Hence, through this study, we aim to investigate the relationship of TB and CNS with nodal involvement and overall prognosis in patients with laryngeal SCCs to further establish the value of TB and CNS as an inexpensive, easily assessable, yet highly reliable prognostic factor.

## 2 Materials and Methods

This is a cross‐sectional retrospective study on surgical laryngectomy specimens of patients diagnosed with laryngeal SCC who underwent surgery between 2016 and 2021 in Amir Alam Hospital. The study included all cases of laryngeal SCC that underwent total laryngectomy and neck dissection. It specifically excluded histologic subtypes of SCC, including basaloid and verrucous SCC, and individuals with distant metastasis at diagnosis. Additionally, patients with a history of prior malignancy and previous exposure to radiotherapy and chemotherapy were not eligible for inclusion in the study.

We gathered patients' ages, genders, tumor sites, and tumor stages from medical records. Due to the retrospective nature of the data collection in this study, there were no specific guidelines for follow‐up. Consequently, the duration of patient monitoring varied, and 36 months of follow‐up status was obtained through phone calls.

The pathology department provided the hematoxylin and eosin‐stained slides of the resected tissue samples. Two pathologists then reviewed all slides to confirm the diagnosis, assess WHO histologic grade, evaluate the presence or absence of TB and CNS, and document other histologic findings such as lymphovascular invasion, perineural invasion, and nodal involvement. Tumor budding, referring to the branching of tumor clusters, is characterized histologically by less than five tumor cells within the stroma/parenchyma. Based on the study of Boxberg et al., TB was assessed in 10 continuous HPFs (magnification 400×) in areas showing maximum budding in the tumor‐invasion‐front and was categorized into low TB and high TB groups, defined as 1–14 budding nests and ≥ 15 budding nests, respectively. CNS was also characterized as clustered tumor cells surrounded by tumor stroma and categorized into four groups as follows: > 15 tumor cells = large nests, 5–15 tumor cells = intermediate nests, 2–4 tumor cells = small nests, and discohesive tumor cells without nested architecture = single‐cell invasion [12].

Statistical analysis was performed by IBM SPSS Statistics 21. Descriptive statistical methods (frequency and percentage distributions) were used to evaluate the quantitative data, and the Chi‐square test was used to compare the qualitative data. Multivariate survival analysis was performed using the Cox regression method. Survival analyses were performed using the Kaplan–Meier method. The results were evaluated with a significance level of *p* < 0.05 and a 95% confidence interval.

## 3 Results

**FIGURE 1:**
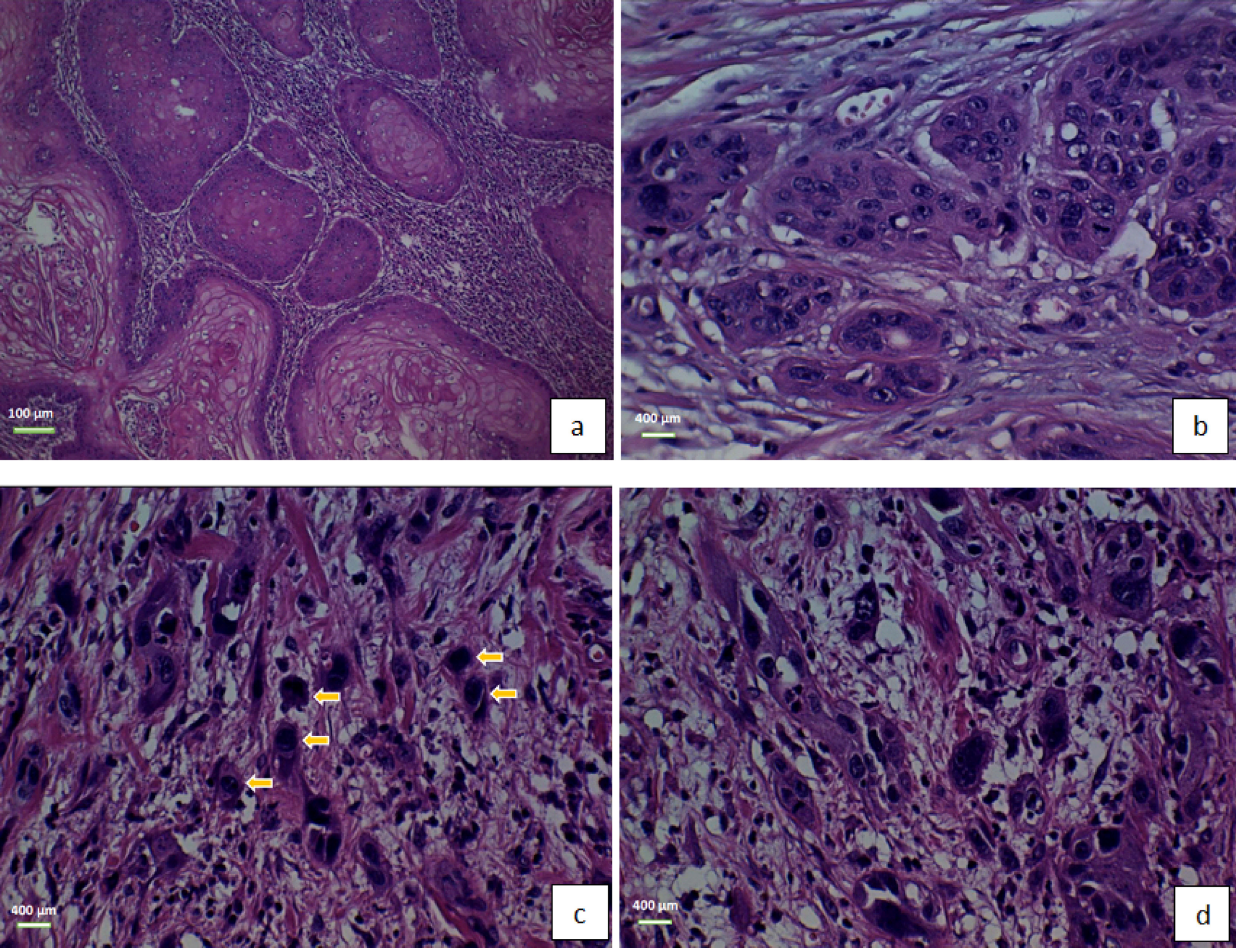
Microscopic examination of laryngeal SCC. No evidence of budding activity, including large cell nest sizes (H&E, ×100) (a), high‐power view of intermediate nests of tumoral cells with no evidence of budding activity (H&E, ×400) (b), high budding activity, and single‐cell infiltration (indicated by arrows) (H&E, ×400) (c), high budding activity, and small cell nest size (H&E, ×400) (d).

This study analyzed 128 cases of laryngeal squamous cell carcinoma, consisting of 118 men (92.2%) and 10 women (7.8%) with an average age of 59.32 ± 7.62 and a median age of 60 years (range: 41–82 years). Clinical staging showed 21 (16.4%) cases of Stage II, 28 cases (21.9%) of Stage III, and 79 cases (61.7%) of Stage IV. Most tumors (75%) were located in the transglottic region, while the remaining 25% were in the supraglottic area. Tumor histologic grade was well‐differentiated in 80 lesions (62.5%), moderately differentiated in 43 lesions (33.6%), and poorly differentiated in 5 lesions (3.9%) based on WHO classification. Positive lymph node involvement, extra‐laryngeal extension, vascular invasion, and perineural invasion were present in 53 (41.4%), 75 (58.6%), 22 (17.2%), and 35 (27.3%) of cases, respectively. During the follow‐up period of 36 months, 48 patients (37.5%) survived, while the remaining 80 cases (62.5%) were deceased. Tumor budding was observed in 86 cases (67.2%), with high budding detected in 35 lesions (27.3%) and low budding in 51 lesions (39.8%). Cell nest size was classified as large in 18 lesions (14.1%), intermediate in 24 lesions (18.8%), small in 47 lesions (36.7%), and single cell in 39 lesions (30.5%) (Figure 1). A summarized outline of the clinical and histological findings can be found in Table 1.

**TABLE 1:** A summary of clinical and histopathological findings.

Tumor findings	Variables	*N* (%)
Tumor location	Transglottic	96 (75%)
	Supraglottic	32 (25%)
Histologic grade	Well‐differentiated	80 (62.5%)
	Moderately‐differentiated	43 (33.6%)
	Poorly‐differentiated	5 (3.9%)
UICC/TNM stage	II	21 (16.4%)
	III	28 (21.9%)
	IV	79 (61.7%)
Extralaryngeal extension	Present	75 (58.6%)
	Absent	53 (41.4%)
Nodal involvement	Present	53 (41.4%)
	Absent	75 (58.6%)
Perineural invasion	Present	35 (27.3%)
	Absent	93 (72.7%)
Vascular invasion	Present	22 (17.2%)
	Absent	106 (82.8%)
Tumor budding	Low‐budding	51 (39.8%)
	High‐budding	35 (27.3%)
	Absent	42 (32.8%)
Cell nest size	Single cell	39 (30.5%)
	Small	47 (36.7%)
	Intermediate	24 (18.8%)
	Large	18 (14.1%)
Survival rate	Disease‐free patients (censored)	48 (37.5%)
	Death (event)	80 (62.5%)

We proceeded to examine how TB relates to various clinical and pathological factors. Our Chi‐square test results indicated a significant correlation between TB and nodal involvement (*p* = 0.015), vascular invasion (*p* = 0.035), and mortality rate (*p* = 0.001). However, TB did not show any correlation with tumor location, histologic grade, clinical stage, extra‐laryngeal extension, or perineural invasion. We also studied the correlation between TB scoring and other variables, which revealed a statistical significance between high TB and extra‐laryngeal extension (*p* = 0.006), clinical stage (*p* = 0.011), and mortality (*p* = 0.001).

Furthermore, we analyzed the correlation of CNS subgroups with other variables. While the size of the nest did not impact perineural or vascular invasion, lesions with small nest size were significantly associated with the clinical stage (*p* = 0.047), extra‐laryngeal extension (*p* = 0.015), and mortality rate (*p* < 0.001). The correlation of tumor budding and cell nest size with other variables can be found in Tables 2 and 3, respectively.

**TABLE 2:** The correlation of tumor budding with clinicopathological findings.

Variable		Low‐budding	High‐budding	*p*
Age		59.13 ± 7.19	60.2 ± 8.11	0.525
Sex	Male	49 (96.1%)	32 (91.4%)	0.365
	Female	2 (3.9%)	3 (8.6%)	
Histologic grade	Well‐differentiated	32 (62.7%)	21 (60%)	0.664
	Moderately‐differentiated	17 (33.3%)	11 (31.4%)	
	Poorly‐differentiated	2 (3.9%)	3 (8.6%)	
UICC/TNM stage	II	9 (17.6%)	2 (5.7%)	0.011
	III	14 (27.5%)	3 (8.6%)	
	IV	28 (54.9%)	30 (85.7%)	
Nodal involvement	Present	22 (43.1%)	20 (57.1%)	0.202
	Absent	29 (56.9%)	15 (42.9%)	
Extralaryngeal extension	Present	26 (51%)	28 (80%)	0.006
	Absent	25 (49%)	7 (20%)	
Perineural invasion	Present	17 (33.3%)	10 (28.6%)	0.640
	Absent	34 (66.7%)	25 (71.4%)	
Vascular invasion	Present	11 (21.6%)	8 (22.9%)	0.887
	Absent	40 (78.4%)	27 (77.1%)	
Survival	Disease‐free patients (censored)	17 (33.3%)	1 (2.9%)	0.001
	Death (event)	34 (66.7%)	34 (97.1%)	

**TABLE 3:** The correlation of cell nest size with clinicopathological findings.

Variable		Single cell	Small	Intermediate	Large	*p*
Age		58.8 ± 8.4	60.21 ± 6.7	60 ± 7.56	87.27 ± 8.2	0.515
Sex	Male	37 (94.9%)	43 (91.5%)	24 (100%)	14 (77.8%)	0.066
	Female	2 (5.1%)	4 (8.5%)	0	4 (22.2%)	
Tumor location	Transglottic	31 (79.5%)	36 (76.6%)	15 (62.5%)	14 (77.8%)	0.465
	Supraglottic	8 (20.5%)	11 (23.4%)	9 (37.5%)	4 (22.2%)	
Histologic grade	Well‐differentiated	23 (59%)	30 (63.8%)	17 (70.8%)	10 (55.6%)	0.285
	Moderately‐differentiated	12 (30.8%)	16 (34%)	7 (29.2%)	8 (44.4%)	
	Poorly‐differentiated	4 (10.3%)	1 (2.1%)	0	0	
UICC/TNM stage	II	2 (5.1%)	9 (19.1%)	4 (16.7%)	6 (33.3%)	0.047
	III	5 (12.8%)	12 (25.5%)	7 (29.2%)	4 (22.2%)	
	IV	32 (82.1%)	26 (55.3%)	13 (54.2%)	8 (44.4%)	
Nodal involvement	Present	20 (51.3%)	22 (46.8%)	7 (29.2%)	4 (22.2%)	0.096
	Absent	19 (48.7%)	25 (53.2%)	17 (70.8%)	14 (77.8%)	
Extralaryngeal extension	Present	31 (79.5%)	23 (48.9%)	13 (54.2%)	8 (44.4%)	0.015
	Absent	8 (20.5%)	24 (51.1%)	11 (45.8%)	10 (55.6%)	
Perineural invasion	Present	13 (33.3%)	14 (29.8%)	4 (16.7%)	4 (22.2%)	0.640
	Absent	26 (66.7%)	33 (70.2%)	20 (83.3%)	14 (77.8%)	
Vascular invasion	Present	8 (20.5%)	11 (23.4%)	2 (8.3%)	1 (5.6%)	0.203
	Absent	31 (79.5%)	36 (76.6%)	22 (91.7%)	17 (94.4%)	
Survival	Disease‐free patients (censored)	2 (5.1%)	16 (34%)	16 (66.7%)	14 (77.8%)	< 0.001
	Death (event)	37 (94.9%)	31 (66%)	8 (33.3%)	4 (22.2%)	

**FIGURE 2:**
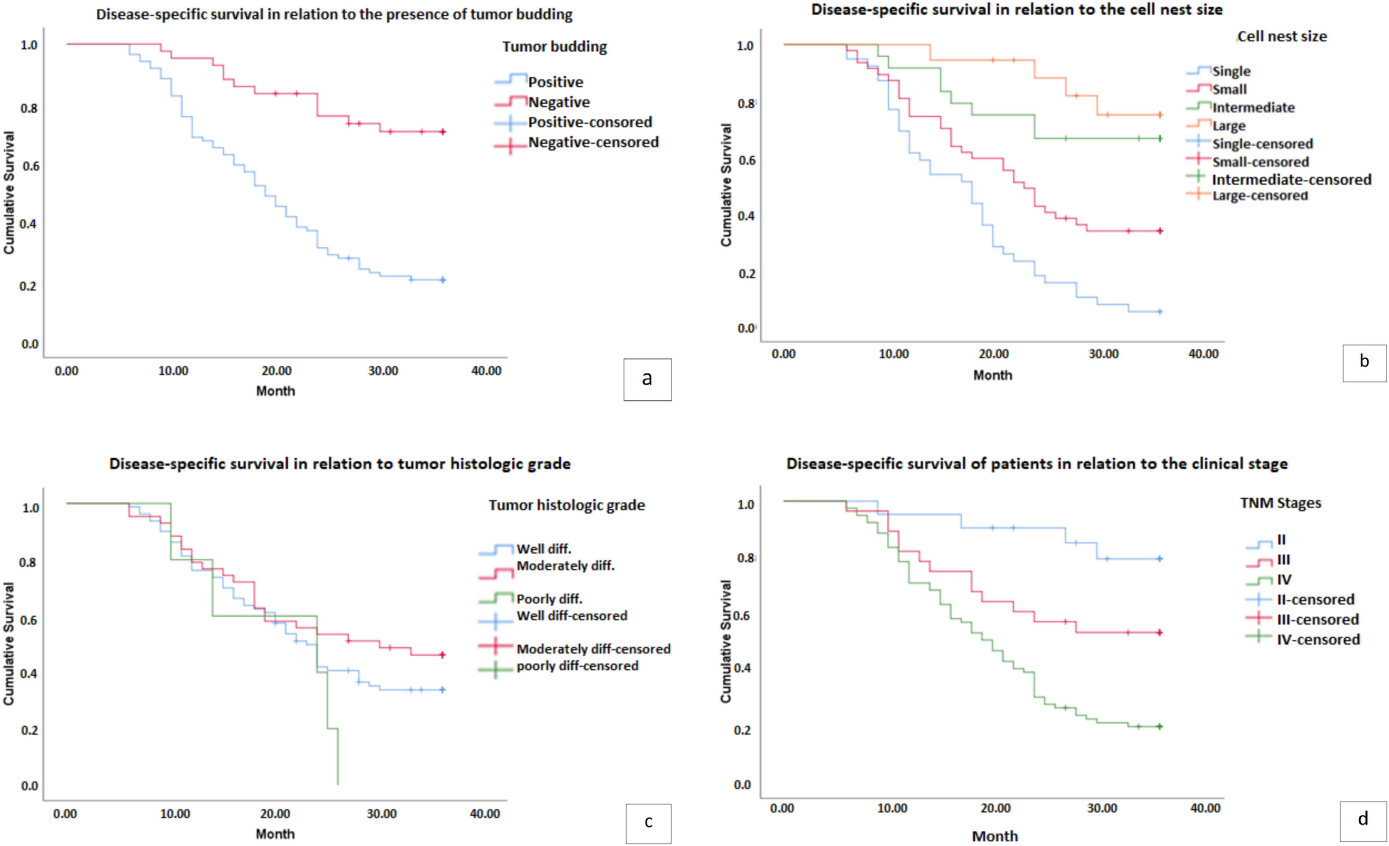
Disease‐specific survival of patients in relation to the presence of tumor budding (a), cell nest size (b), tumor histologic grade (c), and clinical stage (d). The correlation between tumor budding (TB), cell nest size (CNS), and clinical stage demonstrated statistically significant associations with the disease‐specific survival (DSS) rate, with *p* values of < 0.001, < 0.001, and < 0.001, respectively. High budding is linked to a notable decrease in DSS (a). Moreover, single‐cell invasion, small CNS, and intermediate CNS exert a statistically significant adverse impact on DSS (b). However, there was no observed correlation between DSS and the histopathologic grade of differentiation, with a *p* value of 0.199 (c).

Our multivariate analysis identified TB, CNS, and clinical stage as independent prognostic factors for mortality rate, summarized in Table 4. Finally, we evaluated the disease‐specific survival (DSS) of patients using the Kaplan–Meier method concerning tumor histological grade, clinical stage, presence or absence of tumor budding, and cell nest size. Apart from histologic grade, our data revealed a statistically significant difference between disease‐specific survival in each group (Figure 2).

**TABLE 4:** The correlation of histopathologic factors with disease‐specific mortality.

Variable	Beta	95% CI	*p*
Tumor budding	0.266	0.026 to 0.307	0.021
Cell nest size	−0.233	−0.218 to −0.005	0.041
Histologic grade	−0.043	−0.154 to −0.082	0.542
Nodal involvement	−0.001	−9.168 to 0.166	0.992
Vascular invasion	−0.075	−0.300 to 0.108	0.352
Perineural invasion	0.002	−0.154 to 0.160	0.972
Extralaryngeal extension	0.029	−0.256 to 0.312	0.196
UICC/TNM stage	0.360	0.038 to 0.422	0.019

## 4 Discussion

### 4.1 Background of Laryngeal SCCs

Despite recent advancements in diagnostic and therapeutic approaches for LSCC, the overall survival rate for this common neoplasm remains modest [17]. Currently, the UICC/TNM staging system is used to determine the prognosis of LSCC by considering factors such as surgical margin status, tumor differentiation, lymphovascular invasion, perineural invasion, and recipient of adjuvant therapy [18, 19]. However, studies have shown that even among patients with the same tumor stage, there is significant discordance in the predictability of routinely reported prognostic factors [12]. Furthermore, upstaging or downstaging occurs in a third of LSCC patients, impacting margin status, therapeutic approaches, and recurrence rate [20]. It is essential to identify an independent prognostic factor to determine which patients are suitable for organ‐preserving treatment, as the impact of total laryngectomy on psychological and functional aspects can be significant [21]. Our research indicates that a significant percentage of cases, over 62%, were classified as Stage IV, despite exhibiting well‐differentiated features in more than 62% of cases. Additionally, although the mortality rate was high at 63%, vascular and perineural invasion were present in less than 30% of cases. These findings highlight the potential discrepancies between clinical and histopathological prognostic factors.

### 4.2 History of Tumor Budding in SCC

Recent studies suggest that a combined TB/CNS‐based grading system holds promise in predicting outcomes for SCC patients across various sites [22]. TB is recognized as an independent prognostic factor in many solid tumors [18] and even preoperative biopsies can provide valuable information about invasion depth, lymph vascular invasion, and invasion patterns [23]. In oral SCC, TB has been shown to negatively impact survival outcomes, regardless of tumor location or pathological stage [24, 25]. Therefore, validating the prognostic significance of TB and CNS is beneficial, as routine H&E‐stained slides can assess them with high interobserver, and intraobserver agreement [26]. However, studies on SCCs of the larynx and hypopharynx are limited, and results vary due to differences in pathological and clinical variables.

Various methods for detecting TB have been suggested, but the scoring system proposed by Boxberg et al. is sufficient for the risk stratification of LSCCs. Boxberg et al. developed their approach based on a validated grading system for esophageal SCC, which incorporates the budding activity score and intratumoral CNS score. TB quantifies the dissociative growth of cancer cells, while CNS represents the highest level of cellular discohesion qualitatively. Combining these two factors provides insight into tumor invasion and can identify early signs of metastasis [27]. This grading system is an independent prognostic factor for patient survival in SCCs of the larynx, hypopharynx, esophagus, oral cavity, and lung [12, 28].

Additionally, several studies have pointed out the limitations of intra‐operative evaluation of tumor budding (TB) and cell nest size compared to formalin‐fixed specimens due to quality inconsistencies and artifacts in cryo‐fixed sections [29]. Consequently, a few fresh laryngectomy specimens were excluded from our study.

While evaluating peripheral TB (PTB) is the most recommended method, assessing intratumoral TB (ITB) also demonstrates a strong correlation with tumor aggressiveness, prognosis, and PTB results, especially in cases with high TB [30]. The significance of ITB is particularly notable in biopsy samples as they may not fully represent the tumor front [31]. High TB, regardless of location, is generally associated with a poorer outcome. However, our study focused specifically on PTB, as ITB is typically viewed as a supplementary factor and is preferred in biopsy and post‐treatment samples [32].

### 4.3 Immerging Importance of Tumor Budding

Our analysis indicates that the presence of TB is significantly correlated with nodal involvement, vascular invasion, and mortality rate. Ekmekci et al. also found a statistical correlation between lymph node metastasis, perineural invasion, and lymphovascular invasion with TB, although their scoring system differed from ours [33]. We found that categorizing TB into low‐budding and high‐budding activity is more practical and reproducible than the three‐tier semiquantitative grading used in Ekmekci's study. However, some studies suggest that the three‐tier system has better prognostic power [34]. The correlation between TB and lymph node metastases was confirmed in the study of Abd Raboh, Mady, and Hakim, who evaluated the predictive value of TB in LSCC using pan‐cytokeratin immunostaining [35]. Similar results were observed in oral, nasopharyngeal, hypopharyngeal, lung, and cervical SCCs [27, 36, 37, 38]. Synchronous increased TB activity and perineural invasion were also reported in oral SCCs [39].

Additionally, TB was considered an independent predictor of regional metastases in patients with clinically node‐negative T1 and T2 oral SCCs [40, 41]. The study by Karpathiou et al. further confirmed the association between TB and advanced T classification [14]. The predictive ability of TB for lymph node metastasis is valuable as routinely reported lymph node status can be underestimated due to inadequate dissection and sampling [42].

Furthermore, there is no agreement on the most effective predictive methods for detecting regional metastases in patients with node‐negative head and neck SCCs in the early stages [40, 43]. As a result, including TB analysis in pathology reports can alert clinicians to patients with a poor prognosis. Elseragay et al. have even proposed that TB analysis can enhance the predictive accuracy of the WHO histopathologic grading system in early oral SCCs.

Our study observed that high TB scores were significantly linked to a greater likelihood of extra‐laryngeal extension and a higher clinical stage. Patients with high TB scores also had a considerably higher death rate compared to those with low TB scores. Similarly, Paşaoğlu et al. reported a substantial decline in disease‐free and overall survival in patients with high TB scores [15]. Additionally, Imai et al. conducted a retrospective analysis of the risk factors for cervical lymph node metastasis in endoscopically resected superficial hypopharyngeal cancers. This study suggested that high tumor budding is a robust predictor of cervical nodal involvement; however, the multivariate analysis did not support a significant correlation between these two factors, potentially due to limitations caused by focusing solely on a specific site and procedure [44].

It is worth noting that the TB score is associated with lymph node metastasis, local recurrence, lymphoid infiltration, and infiltrative pattern of invasion, which has been validated in oral SCCs [45]. Caruntu et al. also discovered a link between high TB scores and larger tumor dimensions as well as advanced clinical stages [39]. As a result, it is recommended to report the TB score for each case to emphasize the significance of implementing a more rigorous follow‐up plan.

### 4.4 Quick Look at Cell Nest Size

Although research on CNS is significantly more limited than TB studies, the literature reports a correlation between single‐cell invasion and a worse prognosis [13, 46, 47]. Our study discovered a significant connection between a smaller CNS, a higher clinical stage, and a more significant extra‐laryngeal extension. We also found that smaller CNS were linked to a higher mortality rate, particularly in cases of single‐cell invasion. These results align with the existing, limited data in the literature. Karpathiou et al. explored the prognostic significance of TB and CNS in laryngeal and hypopharyngeal SCCs, tumor‐stroma ratio, and stroma type. Their data confirms the adverse prognostic effect of smaller CNS accompanied by poorer lymphocytic reaction, which indicates the relation of tumor microenvironment with cancer progression [14, 48]. Tan and Taskin found the association of perineural invasion and stage with CNS, in addition to the significance of TB in predicting overall survival. They suggested adding TB and CNS to pathologic reports as histopathologic biomarkers that indicate a higher probability of recurrence, metastases, and poor survival [49].

### 4.5 TB/CNS Significance on Prognosis

Our multivariate analysis indicates that TB, CNS, and clinical stage are independent prognostic factors for mortality. This aligns with prior research that indicates high TB and smaller CNS are linked to adverse outcomes in oral SCC [50]. Sarioglu et al. have also suggested that TB is a valuable prognostic factor for distant‐metastases‐free survival [51]. Likewise, Jesinghaus et al. have confirmed the prognostic impact of TB and CNS on overall, disease‐free, and disease‐specific survival independent of clinical stage in esophageal SCCs. They have also demonstrated the efficacy of TB and CNS in predicting the depth of infiltration and nodal involvement in biopsy samples [52]. Yadav et al. have identified TB as an independent poor prognostic marker in oral SCCs exhibiting de‐differentiation, loss of adhesion, and epithelial‐to‐mesenchymal transformation [53].

Our study has further revealed that TB is associated with a significant decrease in disease‐specific survival (DSS). Additionally, single‐cell invasion, small CNS, and intermediate CNS have a statistically significant negative effect on DSS. These results, together with the findings reported in the literature, support the recognition of TB and CNS as independent risk factors for poor DFS in LSCC [16]. High TB has also been correlated with shorter DFS and reduced overall survival in head and neck and oral SCC [35]. Clinical stage is another associated factor with DSS.

However, our study found no correlation between DSS and the histopathologic grade of differentiation. The impact of histologic grade on the survival of LSCC patients remains a topic of debate, with some studies confirming its association with tumor aggressiveness and metastases, while others find the relationship to be unclear [54, 55]. To clarify this ambiguity, a larger population matched‐pair analysis could aid in more accurately comparing survival rates across different histologic grades.

### 4.6 Our Strengths and Weaknesses

Considering everything, the strength of our study relies on the synchronous evaluation of TB and CNS and their correlation with other prognostic factors. Our study adds weight to the adequacy of the simple, reliable, reproducible, and cost‐effective TB/CNS grading system. However, limitations include the relatively short follow‐up period and limited sample size for certain controversial prognostic variables, like tumor site and histologic grade.

### 4.7 Conclusion

Despite its limitations, our study supports the potential role of TB and CNS in determining the prognosis of LSCC. The two‐parameter scoring system used in our study is easily evaluated during basic histopathologic examination and does not require additional ancillary or biomolecular studies. While differences in parameters, follow‐up duration, and study designs may confound the results of our study and previous ones, the overall conclusion underscores the importance of the TBNS grading system in LSCC. Given its accessibility and low interobserver variability, including TBNS scores in pathology reports could facilitate individualized risk assessment and treatment planning. Several studies have validated the association of TB with lymph node metastasis and overall survival, highlighting the detrimental impact of higher TB. Hence, the presence of TB can significantly influence the postoperative adjuvant therapy, assessment of metastases, and the scheduling of patient follow‐up. When considered in conjunction with CNS, TB exceeds the prognostic capabilities of the WHO system, demonstrating substantial prognostic potential that demands attention.

Therefore, integrating TBNS scores into pathology reports can aid personalized patient care regardless of the scoring method.

## Author Contributions

**Kiana Anousha:** conceptualization (equal), methodology (equal), validation (equal), writing – original draft (equal). **Mohammad Moin Shekari:** conceptualization (equal), data curation (equal), resources (equal), software (equal), validation (equal). **Elham Mirzaian:** formal analysis (equal), investigation (equal), methodology (equal), project administration (equal), supervision (equal). **Tahere Yousefi:** conceptualization (supporting), formal analysis (equal), methodology (equal), software (equal), writing – original draft (equal). **Amin Beheshti:** data curation (equal), formal analysis (equal), methodology (equal), resources (equal), software (equal). **Maryam Lotfi:** data curation (equal), investigation (equal), project administration (equal), supervision (equal), writing – review and editing (equal).

## Ethics Statement

The Ethics Committee of the Tehran University of Medical Sciences by the code IR.TUMS.AMIRALAM.REC.1401.032 has approved this study.

## Conflicts of Interest

The authors declare no conflicts of interest.

## Data Availability

The datasets that were generated and examined during the current research are not publicly available. However, interested parties may obtain access to the datasets by making a reasonable request to the corresponding author.
